# Effect of an Accidental Colchicine Overdose in a COVID-19 Inpatient With Bilateral Pneumonia and Pericardial Effusion

**DOI:** 10.7759/cureus.35909

**Published:** 2023-03-08

**Authors:** Tsanko Mondeshki, Radoslav Bilyukov, Vanyo Mitev

**Affiliations:** 1 Department of Propaedeutics of Internal Diseases, University Hospital Alexandrovska, Medical University-Sofia, Sofia, BGR; 2 Department of Chemistry and Biochemistry, Medical University-Sofia, Sofia, BGR

**Keywords:** hymecromone, bromhexine, cytokine release storm, colchicine, covid-19

## Abstract

A 32-year-old patient with COVID-19 pneumonia and pericardial effusion mistakenly took 15 mg of colchicine over 10 hours. He developed diarrhea that resolved two days after colchicine was stopped. Remarkably, this single overdose of colchicine, without any additional therapy, resulted in the complete recovery of bilateral pneumonia and pericardial effusion, and the patient was discharged on the hospital day 9th.

This case demonstrates the possibility that high colchicine doses may have a major role and a dramatic effect in the treatment of COVID-19 patients.

## Introduction

Presentation of COVID-19 infection ranges widely from a fairly mild case with low-grade fever and cough to severe disease leading to acute respiratory distress syndrome (ARDS), coagulopathy, multisystem organ failure, and death. About 15% of the cases develop severe disease with complications and are hospitalized [1]. Mortality in COVID-19 patients has been associated with the so-called cytokine storm (CS). CS is a severe immune reaction (hypercytokinemia - e.g., IL-1/6/8/10/1RA, TNF-α (tumor necrosis factor-α), CXCL10, etc.), leading to an increased risk of vascular hyperpermeability, multiorgan dysfunction, and eventually death. Normally, cytokines are part of the body's immune response to infection, but their uncontrolled, excessive, and sudden release in large quantities (CS) can cause multi-organ injury and death [2]. It is now well established that the cause of CS is the aberrant activation of the NLRP3 (Nod-like receptor family, pyrin domain-containing 3) inflammasome [2]. The main structural protein of SARS-CoV-2 (N-protein) interacts directly and induces hyperinflammation of NLRP3 inflammasome aggravating lung injury and accelerating death in mouse models [3]. A number of other mechanisms of NLRP3 inflammasome activation by SARS-CoV and SARS-CoV-2, including via ORF3a protein, ORF8b protein, Ang II accumulation, Ca2+ transport through the SARS-CoV E protein channel have been described [2]. The central role of NLRP3 in severe COVID-19 makes it a major therapeutic target. A tubulin inhibitor and a microtubule disrupting agent colchicine is a medication approved for the treatment of gout, Behçet's disease, recurrent pericarditis, and familial Mediterranean fever. It causes inhibition of neutrophil chemotaxis, adhesion, and mobilization, blocks NLRP3 inflammasome oligomerization and thus prevents the release of active IL-1β and respectively inhibits the effects of downstream cytokines, i.e. IL-6, TNF-α reduction, etc. This makes colchicine a promising anti-COVID-19 drug [4]. Many observational studies and randomized clinical trials were initiated to test this hypothesis, leading to conflicting results on the colchicine effect [5]. The recent clinical study (NCT04375202) of Carlo Perricone et al., who was among the early advocates of the positive effect of colchicine, seemed to put an end to the controversy, not confirming the curative effect of colchicine at the standard colchicine doses [6].

Our treatment regimen during the pandemic was adjusted to include high doses of colchicine since our experience showed that the standard doses had no effect. The therapeutic dosage of colchicine in our adjusted treatment regimen is calculated by the formula (0.5 mg per every 10 kg) - 0.5 mg loading daily dose, which should not exceed 5 mg. The maintenance dose is half the loading dose [7,8]. This prevents CS, by decreasing the level of inflammatory markers, including IL-6, C-reactive protein (CRP), and D-dimers, and most importantly reduces the mortality between four and five times of inpatients with COVID-19 [9]. Here we describe the therapeutic effect of an accidental colchicine overdose on a COVID-19 inpatient with bilateral pneumonia and pericardial effusion.

## Case presentation

A 32-year-old unvaccinated patient was admitted to the Infectious Diseases Department with complaints of persistent fever up to 38.5°C, joint pain, and general fatigue for the last week. He has been treated as an outpatient with Levofloxacin 500 mg for six days without effect. He did not report past and accompanying illnesses, he did not report allergies to food and medications. He was employed. At the time of patient hospital admission (26.10.21) the dominant variant of SARS-CoV-2 in Bulgaria was Delta.

The physical examination revealed a moderately impaired general condition, height 182 cm, body weight 85 kg, and BMI 25.7. Body temperature up to 37.4°C at the time of examination. Chest - symmetrical. Bilateral vesicular breathing with added crepitations in both lung bases. Rhythmic heart activity, heart rate 79/min, without pathological noises. Arterial pressure 130/80 mmHg.

On admission, a rapid antigen test and polymerase chain reaction (PCR) for SARS-CoV-2 were performed, which were positive.

Laboratory tests demonstrated slightly increased values of inflammatory markers - CRP, ferritin, as well as an increased value of D-dimer. Blood gas analysis shows hypoxemia with hypocapnia and respiratory alkalosis (Table 1).

**Table 1:** Laboratory results PCR: polymerase chain reaction; MCV: mean corpuscular volume; MCH: mean corpuscular hemoglobin; MCHC: mean corpuscular hemoglobin concentration; PDW: platelet distribution width; INR: international normalized ratio; aPTT: activated partial thromboplastin clotting time; CRP: C-reactive protein

Blood tests	Range	26.10.2021 21:40	29.10.2021 14:31	03.11.2021 07:20	05.11.2021 12:40	2.12.2021 11:30
PCR test COVID-19 (SARS-CoV-2-RNA)		(+) positive				(-) negative
Erythrocytes (Er) 10^12/L	4.4-5.9	5.3	5	5.3	5.5	5.2
Haemoglobin (Hb) (g/L)	135-180	155	146	152	161	169
Haematocrit (Ht) (%)	0.4-0.53	0.45	0,43	0.44	0.47	0.51
MCV (fL)	82-96	84	86	83	86	99
MCH (pg)	27-33	29	29	29	29	32
MCHC (g/L)	300-360	348	338	348	340	329
Thrombocytes (Tr) (10^9/L)	130-440	298	256	241	210	263
PDW (%)	8-23	11.9	13,3	12.6	13.1	15.5
Mean platelet volume (MPV) (fL)	6.1-11.5	10.3	10,7	10.7	10.8	11.8
Leukocytes (Leu) (10^9/L)	3.5-10.5	4,3	16.4	25	33,1	8.3
Ne% - Neutrophilic granulocytes	40-70	47		85	73	65
NE# - Neutrophilic granulocytes-number	2-7	2.0		21.2	24.0	5.4
LY% - Limphocytes	20-48	39		7	15	8.5
LY# - Lymphocytes granulocytes - count	1-4	1.7		1.9	4.9	1.9
EO% - Eosinophilic granulocytes	0-6.5	0.2		0.0	0.8	3.7
EO# - Eosinophilic granulocytes - count	0-0.5	0.0		0.0	0.3	0.3
BA% - Basophils granulocytes	0-2	0		1	0	1
BA# - Basophils granulocytes - count	0-0.14	0.01		0.16	0.02	0.04
MO% - Monocytes granulocytes	0-11	14.1		7.2	11.7	8.5
MO# - Monocytes granulocytes - count	0-0.8	0.6		1.8	3.9	0.7
IG (%) - Young granulocytes - %	0-5	0.2		6.4	14.2	0.0
IG # - Young granulocytes - count	0-0.7	0.0		1.6	4.7	0.4
INR	0.8-1.2	1.0	1.1		1.0	
aPTT	27.2-32	32.7	29.1		21.4	
D-dimer (mg/L)	0-0.55	0.59	0.27		0,29	
fibrinogen F-I (g/L)	2-4	3.6	2.3	2,1	2,5	
Bilirubin direct (µmol/L)	0-6.5		2.2			
Uric acid (µmol/L)	200-420			152	203	
BUN (blood urea nitrogen) (mmol/L)	2.5-8.3	3.2	3.5	3.9	3,8	
Total protein - serum (g/L)	64-83	78	66		72	
Albumin serum (g/L)	35-52	46	40	42	41	
Alpha-amylase serum (U/L)	0-100			54	60	
Creatine phosphokinase - serum (CPK) (U/L)	0-190	86	95			
Creatine phosphokinase - MB - serum (U/L)	0-25	14	24			
Sodium serum (mmol/L)	136-151	136	147	142	138	
Potassium serum (mmol/L)	3.5-5.6	4.09	4.42	4.68	5.35	
Chloride serum (mmol/L)	96-110	98	101	105	99	
Alanine aminotransferase (ALAT) - serum (U/L)	0-41	15	20	132	113	36
Aspartate aminotransferase (ASAT) - serum (U/L)	0-40	24	22	16	27	31
Creatinine serum (µmol/L)	62-106	85	68	63	79	84
Lactate Dehydrogenase (LDH) - serum (U/L)	240-480	237	225	206	241	
Glucose serum (mmol/L)	3.6-6.1	5.3	7.2	7.7	4.3	5,1
ph	7.35-7.45	7.53			7,42	
pCO2 (kPa)	4.67-6	2.92			5,02	
pO2 (kPa)	13.окт	8,5			10,3	
Standart serum bicarbonate (SB - mmol/L)	21-25	25.20			24,1	
BE (w)	-2.5-2.5	-3.00			-2,7	
O2 sat %	94-98	90			94	95
tCO2	20-27	18.20			21,8	
Procalcitonin (ng/mL)	0-0.05			0.028		
CRP (mg/L)	0-5	11.2	7,3	0.5	0.2	1,2
Erythrocyte Sedimentation Rate (ESR) (mm/h)	0-20	31	35	38	38	7
Ferritin serum (µg/L)	30-400	798.8	810,5	846.3	852,8	351

**Figure 1:**
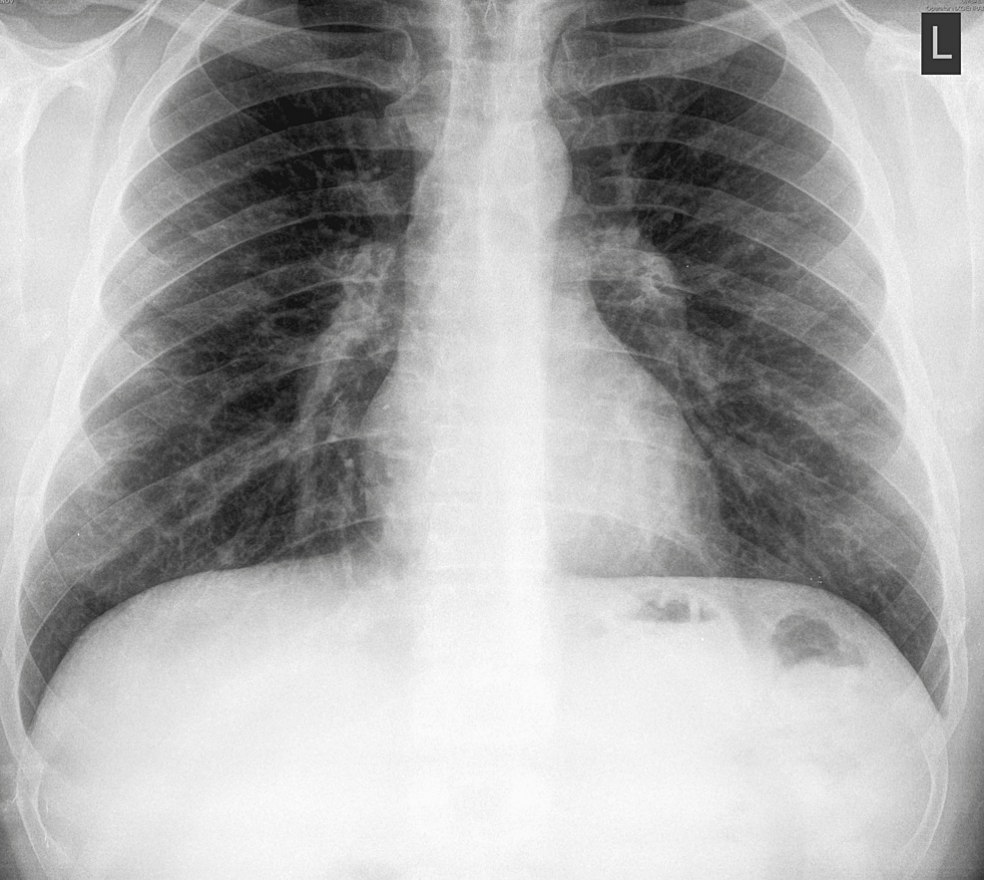
Chest X-ray of the patient upon hospital admission The image demonstrates fine peripheral areas of ground glass opacities with central reticular interstitial thickening.

**Figure 2:**
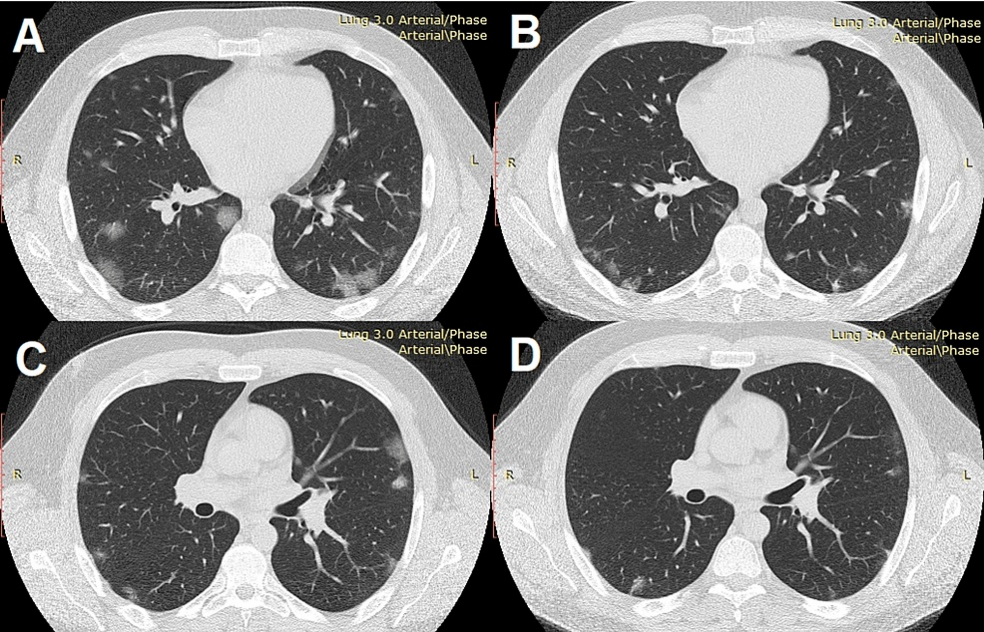
Dynamics of radiological changes in chest CT A and C - axial views with bilateral patchy ground-glass opacities and pericardial effusion B and D - axial views five days after initiation of therapy with reduction of pericardial effusion and ground-glass opacities CT: computed tomography

The patient underwent single view, anteroposterior chest X-ray (Figure 1), and computed axial tomography of the chest (CAT) with evidence of "ground glass" type changes bilaterally, mainly with dorsobasal and subpleural localization (Figures 2A-2C). Data on pericardial effusion - up to 8 mm. Trachea and main bronchi - passable to their subsegmental branches.

Conclusion

Pulmonary changes have the CT morphology of a COVID-19 infection in the exudative phase with involvement of 5-25% of the lung parenchyma (Lung Severity Score: 2).

The initial therapy of the patient included Ceftriaxone (2 g. i.v.); Enoxaparin (0.4 ml/day s.c.); normal saline; Methylprednisolone (40 mg + 20 mg/day i.v.); Bromhexine was administered 3x1 inhalation with nebulizer per day (2 ml Bromhexine (2mg/ml) + 2 ml normal saline); Colchicine - according to the formula ((0.5 mg per every 10 kg) - 0.5 mg), taking one pill every hour for the first three hours, and the rest distributed at equal intervals up to 24 hours.

Due to a misunderstanding, the patient started taking three tablets of colchicine every hour for 10 hours, instead of the prescribed dose above. The total accepted dose for this period was 30 tablets of 0.5 mg or 15 mg. After establishing the mistake made by the patient, the overall therapy was stopped due to the unknown drug interactions and risk of organ damage from a colchicine overdose. Normal saline was administrated 500 ml/four times a day together with Furosemide (ampules - 10mg/ml, 2 ml) one ampule two times daily.

On the seventh day of the stay, a control CT scan was performed, as compared to the previous examination from 27-10-2021. A clear X-ray morphological dynamics was established, expressed in the transformation of the morphological changes of the "frosted glass" type into the so-called irregular pavement type and limited within the involved lung areas with small parenchymal consolidations.

Insignificant lymphadenomegaly persisted in markedly axillary and retrocaval regions, with the largest nodules reaching 7.0 mm. The described pericardial effusion was significantly reduced. Pleural compactions and pleural effusions were no longer visualized. The investigated volume of the upper abdominal floor was of usual CT morphology.

Conclusion

Pulmonary changes are associated with SARS-CoV-2 in the subacute and chronic phases of the disease. A significant reduction in the volume of lung involvement and a reduction in pericardial effusion was observed (Figures 2B-2D).

The patient was released from the hospital and 27 days later control laboratory tests were conducted, which were within the reference limits.

This clinical case demonstrates that the intake of a toxic dose of Colchicine (due to an error on the part of the patient in the course of SARS-CoV-2 virus infection with the development of bilateral pneumonia), led to reverse the development of the X-ray morphological changes within five days without any additional therapy. The monitored pathologically changed laboratory indicators show a reverse development, with the inflammatory markers normalizing in a very short period.

In the other two outpatients' cases, women aged 68 and 62, respectively, took five tablets of 0.5 mg five times for the first 24 hours, achieving a total dose of 25 tablets (12.5 mg), without evidence of pneumonia. The outpatients' symptoms resolved within three days. In all three patients, a lower dyspeptic syndrome was observed, controlled with symptomatic medicines for 24 hours. In all three patients, treatment with colchicine was started after filling informed consent in accordance with the rules of good clinical practice.

## Discussion

In the described cases of colchicine overdoses, the interesting thing is not that they survived without severe side effects, but the accelerated recovery from COVID-19. Our clinical experience with COVID-19-related pneumonia and pericardial effusion showed that the treatment and resolution of the symptoms take much longer in an outpatient setting. The effect of colchicine overdose in the present case confirms our hypothesis, that only high-loading and maintenance doses of colchicine can have a pronounced therapeutic effect [7-9].

**Figure 3:**
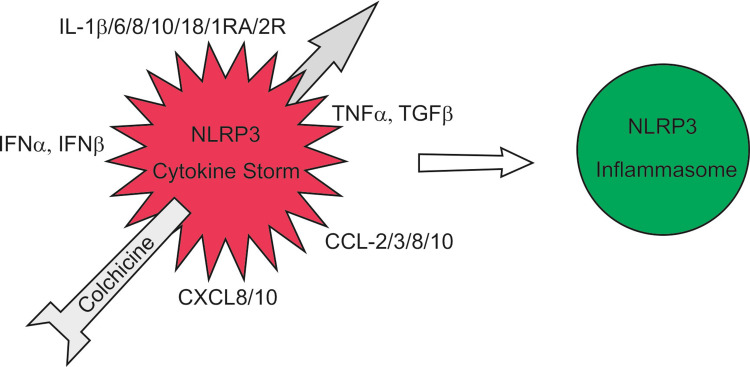
Colchicine prevents the cytokine storm by blocking the hyperactivated NLRP3 inflammasome NLRP3: nod-like receptor family, pyrin domain-containing 3; TNFα: tumor necrosis factor-alpha; TGFβ: transforming growth factor-beta; IFNα: interferon-alpha; IFNβ: interferon-beta

Since CS is considered a major cause of COVID-19 complications and death, our therapeutic strategy targets the inhibition of NLRP3 inflammasome. This will cause the levels of all the cytokines activated by the hyperactivated NLRP3 inflammasome to fall and renders it useless to inhibit the cytokines individually (Figure 3).

Therefore, inhibition of the NLRP3 inflammasome makes administration of tocilizumab and sarilumab (anti-interleukin-6 antibodies), anakinra and canakinumab (IL-1 receptor antagonists), JAK inhibitors, etc. [10] does not make sense.

The optimal dose of colchicine for COVID-19 infection has yet to be established [10]. However, we believe that it is completely pointless to continue clinical research with the so-called standard doses of a maximum loading dose of 1.5 mg followed by 0.5 mg in 60 minutes and a maintenance daily dose that does not exceed 1 mg [5].

We have previously shown a five-fold decrease in the mortality rate when our therapeutic regimen with colchicine was used in inpatient settings [8,9]. (Four hundred and fifty-two inpatients were treated with SOC (standard-of-care) + colchicine); 213 inpatients were treated with SOC (control); RR (0.23, 95% CI 0.136-0.387); OR (0.23, 95%CI = 0.1296-0.4068) p< 0.0001.)

A number of cases of overdose of colchicine are known in the literature. Thus, in the first randomized controlled trial on colchicine in acute gout on day 1 the mean dose of colchicine was 6.7 mg [11]. Terkeltaub RA et al. conducted a clinical trial in 2010 testing the efficacy and safety of high-dose colchicine (4.8 mg) versus low-dose colchicine (1.8 mg) or placebo for the treatment of acute gout flares [12]. This trial, called AGREE trial, demonstrated that low-dose colchicine was as effective as high-dose colchicine for the treatment of gout flares [12]. In familial Mediterranean fever colchicine can be used up to a daily dose of 2 mg in children and 3 mg in adults, or “the maximum tolerated dose if this cannot be appropriate” [13].

We and others think that the findings of AGREE trial may be among the main reasons why all clinical studies testing the therapeutic role of colchicine for COVID-19 patients used low doses, even though AGREE trial itself was designed to test the efficacy of colchicine in acute gout [5]. But gout is not COVID-19!

As we have noted previously, attempts to prove an effect at low doses of colchicine continue to this day, trying to refute "Einstein Insanity": "Insanity is doing the same thing over and over and expecting different results" [8]. Another important methodological limitation of the conducted studies is that the body weight of the patients has not been taken into account. It is not the same to give a 2 mg loading dose to a 50 kg patient and to a 100 kg patient. We have avoided these therapeutic issues by applying our treatment formula [7-9].

At least 43 observational studies and randomized clinical trials have been published so far testing the effect and role of colchicine in the treatment of COVID-19; however, the results of these studies have been controversial and far from conclusive [5,6]. One common methodological feature of these studies is that they use a low-loading dose, not exceeding 2 mg daily [5,9].

The therapeutic range of colchicine is narrow and lies between 0.015 and 0.03 mg/kg [14]. Toxicity typically occurs in doses higher than 0.5 mg/kg/d [15] considering the lethal dose to be 0.8 mg/kg [16]. However, there is no clear-cut line between nontoxic, toxic, and lethal doses of colchicine in humans [15] and the lowest lethal doses have been reported between 7 and 26 mg [17]. In contrast, massive colchicine overdoses i.e. 1.125 mg/kg [18] or 1.38 mg/kg [19] have been reported to lead to recovery. These levels are clearly higher than the fatal ones.

The classic and often cited death case with a dose of colchicine of 7 mg is from 1947. It should be taken into account, however, that it was taken simultaneously with mercury pills [20]. In general, toxicity in doses lower than 0.5 mg/kg/d is usually due to drug interactions or in association with renal impairment [15].

We have a follow-up of about 1,500 COVID-19 inpatients and thousands of outpatients treated with high-dose colchicine. We showed that the mortality in inpatient settings (n=452) was 3.8-4.3% when our treatment regimen with higher doses of colchicine was used compared to the mortality of 18.3% in control cases of standard therapeutic regimens [8,9].

Colchicine higher than standard doses leads to diarrhea, however, this is a very small price to pay for saving a life.

## Conclusions

Colchicine overdose cases demonstrate its therapeutic effect on COVID-19. When given early, it prevents the CS by blocking the NLRP3 inflammasome. Expectedly, diarrhea was the most common side effect, but none developed intoxication at these doses. However, for some of the COVID-19 patients who are prone to develop CS, the choice is high doses of colchicine and diarrhea but alive or dead without diarrhea.
